# Organogermanium, Ge-132, promotes the clearance of senescent red blood cells via macrophage-mediated phagocyte activation

**DOI:** 10.1016/j.heliyon.2023.e23296

**Published:** 2023-12-03

**Authors:** Tomoya Takeda, Junya Azumi, Mika Masaki, Takae Nagasawa, Yasuhiro Shimada, Hisashi Aso, Takashi Nakamura

**Affiliations:** a Asai Germanium Research Institute Co., Ltd., 3-131, Suzuranoka, Hakodate, Hokkaido, 042-0958, Japan; b Laboratory of Animal Health Science, Graduate School of Agricultural Science, Tohoku University, 468-1, Aramaki aza, Aoba, Sendai, Miyagi, 980-8578, Japan

**Keywords:** Organogermanium, RBCs, Macrophage, Phagocytosis, Antioxidant effects

## Abstract

Red blood cells (RBCs) are renewed in a cyclic manner. Aging RBCs are captured and degraded by phagocytic cells, and heme metabolic pigments are subsequently excreted in feces. We evaluated the effect of an organogermanium compound on RBC metabolism and found that the phagocytosis of RAW264.7 macrophage-like cells was increased by treatment with 3-(trihydroxygermyl)propanoic acid (THGP). Additionally, consumption of Ge-132 (a dehydrate polymer of THGP) changed the fecal color to bright yellow and increased the erythrocyte metabolic pigment levels and antioxidant activity in feces. These data suggest that Ge-132 may activate macrophages in the body and promote the degradation of aged RBCs. Furthermore, Ge-132 intake promoted not only increases in RBC degradation but also the induction of erythroblast differentiation in bone marrow cells. The normal hematocrit levels were maintained due to the maintenance of homeostasis, even though Ge-132 ingestion increased erythrocyte degradation. Therefore, Ge-132 enhances the degradation of senescent RBCs by macrophages. In turn, RBC production is increased to compensate for the amount of degradation, and RBC metabolism is increased.

## Highlights

• Ge-132 enhances the erythrophagocytic activity of macrophages.
• Ingestion of Ge-132 increases fecal antioxidant capacity.
• Ge-132 intake promotes the induction of erythroblast differentiation in bone marrow cells.
• Ingestion of Ge-132 promotes the breakdown of RBCs and induces the production of RBCs.

## Graphical abstract

**Figure:**
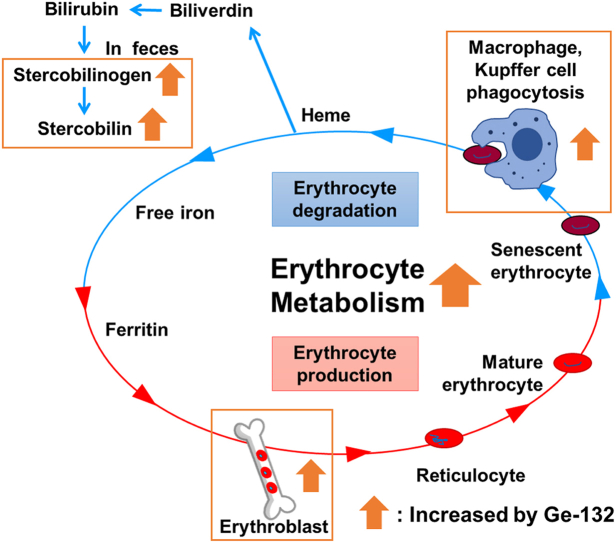

## 1 Introduction

Red blood cells (RBCs) are important cells that carry oxygen throughout the body [1]. However, RBCs are constantly exposed to various stresses, such as oxidative stress, osmotic shock, and capillary pressure. As a result, erythrocytes undergo biochemical and structural changes due to the accumulation of stress-induced damage, and their functions, such as oxygen-carrying capacity, are impaired [2]. Senescent erythrocytes with reduced function are phagocytosed by macrophages and Kupffer cells in the spleen and liver [3,4]. In general, mouse and human erythrocytes have a life cycle of approximately 55 and 120 days from differentiation from hematopoietic stem cells in the bone marrow to decomposition by phagocytosis respectively [5,6]. Regarding the hematopoietic function of erythrocytes, the renal release of erythropoietin is enhanced under hypoxic conditions such as anemia, which promotes the differentiation of erythroblasts in the bone marrow and increases the number of erythrocytes in the blood [7]. However, aging reduces hematopoietic function and increases the proportion of aged erythrocytes in the blood, which is presumed to lead to reduced erythrocyte function and senile anemia [8,9]. In addition, aged RBCs lose their deformability and become prone to agglutination, which has been suggested to be a factor causing blood circulation disorders [2]. Impaired blood flow reduces oxygen and nutrient delivery throughout the body and causes various diseases [10,11]. Therefore, suppression of RBC senescence is important for disease prevention.

Poly-*trans*-[(2-carboxyethyl)germasesquioxane] (Ge-132) is a polymer of 3-(trihydroxygermyl)propanoic acid (THGP; (HO)_3_GeCH_2_CH_2_COOH). It was synthesized in 1967 for human use, and its structure was elucidated by Tsutsui et al., in 1976 [12]. Ge-132 has been confirmed to be a safe substance in several toxicological studies [[13], [14], [15]]. Recently, Asaigermanium_®_ (AG: Ge-132 manufactured at Asai Germanium Laboratory) has been certified as the Japanese version with a self-affirmed generally recognized as safe (GRAS) designation in Japan, and its safety for use as a food has been objectively guaranteed. AG products whose safety has been confirmed are used as ingredients in some foods and cosmetics in Japan. In the United States, supplements with a Ge-132 structure, whose safety has not been confirmed, are distributed on the market as nutritional supplements. To date, Ge-132 has been reported to exert immunostimulatory effects, such as antitumor effects, induction of IFNγ production, and activation of NK cells and macrophages [[16], [17], [18]]. In addition, Ge-132 exerts antioxidant [[19], [20], [21]], anti-inflammatory [22,23] and analgesic effects [24].

We have previously reported that the color of animal and human feces changes to yellow after ingestion of Ge-132 [25]. The major contributors to stool color are the metabolic pigments of heme [26]. Nakamura et al. [25] showed that Ge-132 ingestion caused a change in stool color due to an increase in the amount of the heme metabolite stercobilin in the cecal contents. Furthermore, an increase in the bile bilirubin content and bile acid excretion was confirmed. Bilirubin is one of the products of erythrocyte pigment degradation [27], and stercobilin is the final product of the oxidation of the bilirubin metabolite urobilinogen by intestinal bacteria [28]. Thus, we hypothesized that Ge-132 ingestion promotes erythrocyte degradation by macrophages and increases erythrocyte pigment excretion in the feces.

Considering a previous report on the activation of macrophages by Ge-132 [18,29], phagocytosis of erythrocytes by macrophages may be enhanced by Ge-132. In a previous study, we also found that Ge-132 ingestion increased the amounts of erythrocyte pigment degradation products in the cecal content, but hematocrit values, which indicate the RBC mass in blood, were maintained [25]. Furthermore, germanium dioxide (GeO_2_), which has structural similarity to Ge-132, is toxic [30,31] but has been reported to promote hematopoietic function [32,33]. In other words, Ge-132 may promote the decomposition of RBCs and simultaneously suppress the decrease in RBC mass by enhancing hematopoietic capacity. Therefore, in this study, we investigated the effect of THGP, a hydrolysate of Ge-132, on RBC phagocytic activity by RAW264.7 mouse macrophage-like cells. Furthermore, we investigated the metabolic pigments from erythrocytes in feces following Ge-132 ingestion. Among heme metabolites, bilirubin and urobilinogen have been reported to be pigments with antioxidant properties; thus, we measured the antioxidant capacity of a fecal pigment extract and evaluated the correlation with the quantitative results for the pigment levels. Finally, to investigate the effect of Ge-132 on hematopoiesis, we examined the effect of Ge-132 intake on the ability of myeloid cells to differentiate into burst-forming unit-erythroid (BFU-E) cells.

## 2 Materials and methods

### 2.1 Animals

Male C57BL/6 J mice and male ICR mice were housed individually in a room with a temperature of 20 ± 3 °C on a 12 h light and dark cycle. The mice were purchased from Japan Charles River (Yokohama, Japan). All animal handling procedures and experimental protocols were conducted in compliance with guidelines approved by the Environmental and Safety Committee of Asai Germanium Research Institute.

### 2.2 Phagocytosis assay

RAW264.7 cells, a murine macrophage line (Riken BRC, Ibaraki, Japan), were cultured with Dulbecco's modified Eagle medium (DMEM) (Nissui Pharmaceutical Co., Ltd., Tokyo, Japan) supplemented with 10 % fetal bovine serum (FBS) at 37 °C in the presence of 5 % CO_2_. The cells were seeded in 6-well plates at a density of 5 × 10^5^ cells/well and treated with 0, 50, 500 and 5000 μM THGP for 1 day. The phagocytic activity was evaluated using a phagocytosis assay kit (Cayman Chemical Company, Ann Arbor, MI, USA). FITC-labeled rabbit IgG latex beads were diluted 1:200 with DMEM and incubated with cells for 1 h. Nuclei were stained with Hoechst 33452 (Dojindo Molecular Technologies, Inc., Kumamoto, Japan). Beads phagocytosed by cells were observed with an inverted microscope (Nikon Eclipse TS100, Tokyo, Japan) equipped with a DS-Fi3 digital camera (Nikon).

### 2.3 Analysis of RBC phagocytosis by macrophages

Seven-week-old male C57BL/6 J mice underwent acclimation and were fed a control powder diet for nine days. After the acclimation period, the mice were randomly assigned to two groups of six mice per group. The experimental groups were as follows: (I) the normal chow diet group and (II) the 0.05 % Ge-132 group. Ge-132 (lot. 006316A, purity >99.9 %), the polymer of THGP, was produced at the Hakodate plant at the Asai Germanium Research Institute Co., Ltd. (Hokkaido, Japan). The mice were provided the appropriate experimental diet for four days. The compositions of the diets are shown in Table 1. During the entire breeding period, the mice had free access to food and water. After being fed each experimental diet for four days, the animals were dissected under anesthesia, and whole blood was collected. The collected blood was centrifuged at 850×*g* for 10 min. The supernatant consisting of the blood plasma was discarded, and the packed cells were suspended in a PBS (−) solution. The cell suspension was centrifuged at 850×*g* for 10 min. The supernatant was discarded, 50 μl of the packed cells were frozen for glutathione measurements, and the remaining cells were resuspended in Alsever's solution (Muto Pure Chemicals Co., Ltd., Tokyo, Japan). The suspension was stored at 4 °C and used the day after dissection to test the phagocytosis of erythrocytes by macrophages. RAW264.7 cells were seeded on coverslips in 6-well plates at a density of 5 × 10^5^ cells/well and treated with or without 500 μM THGP for 1 day. Stored blood cells were centrifuged at 300×*g* for 10 min, and the supernatant was discarded. The precipitate was suspended in PBS, and the cell suspension was centrifuged at 300×*g* for 10 min. The precipitate was resuspended in PBS, and the number of RBCs was counted and adjusted to 5 × 10^7^ cells/ml. The prepared erythrocyte suspension solution was added to RAW264.7 cells at a density of 5 × 10^6^ cells/well, and the cells were cultured with 5 % CO_2_ at 37 °C for 1 h. After incubation, the cell surface was washed with PBS to remove excess RBCs. May–Grunwald Stain Solution (Wako Pure Chemical Industrial Co., Ltd., Osaka, Japan) was added, and the cells were stained for 5 min at room temperature (RT). The staining solution was discarded, and the cells were treated with 1/15 M PBS pH 6.4 (Wako Pure Chemical Industrial Co., Ltd.) for 5 min at RT. After the PBS solution was discarded, the cells were stained with 25-fold diluted Giemsa staining solution (Wako Pure Chemical Industrial Co., Ltd.) for 15 min at RT. After the staining solution was discarded, deionized water was added to the cells, and the cells were incubated for 5 min at RT. The stained cells were mounted with 50 % glycerol (Wako Pure Chemical Industrial Co., Ltd.) and observed with an inverted microscope. From each slide, five microscopic fields were randomly photographed with a microscope at 200 × magnification. Approximately 100 RAW264.7 cells were counted, and the numbers of erythrocytes phagocytosed and phagocytic RAW264.7 cells were counted. The counts of phagocytosed RBCs per 100 macrophages were calculated by dividing the number of phagocytosed erythrocytes by the number of RAW264.7 cells and multiplying by 100. The average number of phagocytosed RBCs in phagocytic macrophages was calculated by dividing the number of phagocytosed RBCs by the number of phagocytic RAW264.7 cells.

**Table 1:** Composition of each diet.

	Chow diet	Ge-132 diet
	**(%)**	
**Casein**	**23**	**23**
**α-Corn starch**	**61.5**	**61.45**
**Corn oil**	**5**	**5**
**dl****-methionine**	**0.3**	**0.3**
**Vitamin mixture**	**1**	**1**
**Mineral mixture**	**4**	**4**
**Cellulose**	**5**	**5**
**Choline**	**0.2**	**0.2**
**Ge-132**	**-**	**0.05**
**Total**	**100**	**100**

### 2.4 Quantitative polymerase chain reaction (qPCR)

RAW264.7 cells were seeded in 6-well plates at a density of 5 × 10^5^ cells/well and treated with or without 500 μM THGP for 1 day. Total RNA was extracted from the cells using Isogen (Nippon Gene, Toyama, Japan) according to the manufacturer's instructions. The mRNA levels of the heme oxygenase 1 (Hmox1) and 2 (Hmox2) genes in the cells were determined using qPCR. The expression of the housekeeping gene ribosomal protein S18 (RPS18) was examined as a reference gene. The cDNA templates were synthesized from the total RNA using Super Script III reverse transcriptase (Thermo Fisher Scientific, Massachusetts, USA). The cDNA samples were amplified with the primer set for each gene and SYBR Premix Ex *Taq*II (Takara Bio Shiga, Japan) using a LightCycler 96 system (Roche Diagnostics GmbH). The forward (f) and reverse (r) primers used in this study were as follows: Hmox1f: gtcaagcacagggtgacaga, Hmox1r: atcacctgcagctcctcaaa; Hmox2f: agcagctcaaaacttcccagc, Hmox2r: caaattcaggtccaaggcattc; and Rps18f: ttctggccaacggtctagacaac, Rps18r: ccagtggtcttggtgtgctga. The temperature cycling program for the reaction was as follows: 95 °C for 30 s followed by 40–50 cycles of 95 °C for 5 s and 60 °C for 30 s.

### 2.5 Western blotting

RAW264.7 cells were seeded in 6 cm dishes at a density of 1.5 × 10^6^ cells/well and treated with or without 500 μM THGP for 1 day. After 24 h, the prepared erythrocyte suspension solution was or was not added at a density of 1 × 10^7^ cells/well to RAW264.7 cells and cultured in the presence of 5 % CO_2_ at 37 °C for 1 h. After incubation, the cell surface was washed with PBS to remove excess RBCs. The cells were dissolved in RIPA buffer. The supernatant was collected after centrifugation at 15,000×*g* for 10 min, quantified using the Bradford method and adjusted to a concentration of 1 μg/μl. Twenty micrograms of protein was separated on SDS‒PAGE gels, after which the proteins were transferred to PVDF membranes (ATTO Corporation Tokyo, Japan). Skim milk (5 %) in TBS-T (Tris-buffered saline with 0.01 % Tween-20) was used as a blocking buffer. The primary antibodies were dissolved in blocking buffer and incubated with the membrane overnight at 4 °C. Secondary antibodies were dissolved in blocking buffer and incubated with the membrane for 1 h at room temperature. Anti-heme oxygenase protein (HMOX-1) (Abcam Ltd., Cambridge, UK) and anti-β-actin (Abcam) antibodies were used as primary antibodies. The primary antibody was diluted to the recommended concentration. The secondary antibody (Abcam) used in this experiment corresponded to the primary antibody. β-Actin was used as an internal standard when quantifying the expression of each protein.

### 2.6 Preparation of feces and blood

While undergoing acclimation for three weeks, five-week-old male ICR mice (Japan Charles River, Japan) were fed a chow diet. After the three-week acclimation period, the mice were randomly assigned to two groups fed the chow diet (n = 8) and 0.05 % Ge-132 diet (n = 8). Furthermore, the mice were each provided an experimental diet for five days. For the last week of the acclimation period and experimental periods, the feces were collected each day and stored at −80 °C. Throughout the breeding period, the mice had free access to food and water. After being fed the experimental diet for five days, the animals were dissected. We dissected animals under isoflurane anesthesia and collected whole blood to measure the hematocrit value.

### 2.7 Extraction and analysis of bilirubin and stercobilin pigments

We ground the collected feces and extracted bilirubin and stercobilin pigments from them with 4 ml of acetate (Wako Pure Chemical Industrial Co., Ltd.)/ethyl acetate (Wako Pure Chemical Industrial Co., Ltd)/diluted water (72:18:10, v/v) containing 50 mg of sodium ascorbate (Wako Pure Chemical Industrial Co., Ltd) for 1 day at 4 °C. After extraction, the mixture was centrifuged at 9600×*g* for 15 min at 4 °C, and the supernatant was collected. The supernatant was filtered with a 0.5 μm membrane filter (ADVANTEC MFS, INC. California, USA). A 20 μl sample was injected onto a high-performance liquid chromatography (HPLC) column. Regarding the HPLC conditions, gradient elution was performed using two mobile phases. The first mobile phase (A) was a mixture of 20 mM citrate buffer (pH 5) (Wako Pure Chemical Industrial Co., Ltd.)/methanol (Junsei Chemical Co., Ltd., Tokyo, Japan)/acetonitrile (Junsei Chemical Co., Ltd.) (6:3:1 v/v), the second mobile phase (B) was a mixture of 20 mM citrate buffer (pH 5)/methanol/acetonitrile (2:9:3 v/v), and the flow rate was 0.75 ml/min. The HPLC gradient conditions are shown in Table 2. The column used in this study was a Mightysil RP18-GPII column (Kanto Chemical Co., Inc., Tokyo, Japan). Detection was performed with a photodiode array at 450 and 490 nm. Bilirubin and stercobilin reagents, which served as standards, were purchased from Wako Pure Chemical Industrial Co., Ltd., and Frontier Scientific, Inc. (Utah, USA), respectively.

**Table 2:** HPLC gradient conditions.

	Mobile phase composition		
**Time (min)**	**Eluent A (%)**	**Eluent B (%)**	**Flow rate (ml/min)**
**0**	**100**	**0**	**0.75**
**5**	**100**	**0**	**0.75**
**10**	**0**	**100**	**0.75**
**27**	**0**	**100**	**0.75**
**33**	**100**	**0**	**0.75**
**Eluent A** = **20 mM citrate buffer (**pH **5)/methanol/acetonitrile (6:3:1 v/v)**			
**Eluent B** = **20 mM citrate buffer (**pH **5)/methanol/acetonitrile (2:9:3 v/v)**			

### 2.8 Extraction and analysis of the stercobilinogen pigment

The fecal stercobilinogen pigment was extracted with 3 ml of 0.5 M acetate buffer (pH 5.0) containing 25 mg of sodium ascorbate for 3 h at 4 °C. The inside of the extraction container was filled with nitrogen gas to prevent oxidation. After extraction, petroleum ether was added to the extraction solution, and the mixture was stirred well and centrifuged at 1200×*g* for 5 min at room temperature. The supernatant was collected, and petroleum ether extraction was repeated twice. Then, Ehrlich's reagent was added to the collected supernatant and reacted with the extracted stercobilinogen. The reaction was stopped by adding saturated sodium acetate and centrifuged at 1200×*g* for 5 min at room temperature. The solution from the lower layer was collected, and the absorbance was measured at 560 nm. Urobilinogen, one of the structural isomers of stercobilinogen, was purchased from Santa Cruz Biotechnology (CA, USA) and used as a standard.

### 2.9 Analysis of radical-scavenging activity (RSA) in feces

The water-soluble antioxidants in the feces were extracted with deionized distilled water for 3 h at 4 °C. The inside of the extraction container was filled with nitrogen gas to prevent oxidation. Then, the extraction solution was centrifuged at 1200×*g* for 5 min at room temperature, and the supernatant was collected as a sample to measure RSA. RSA was measured using the 1,1-diphenyl-2-picryl-hydrazyl (DPPH) (Wako Pure Chemical Industrial Co., Ltd.) assay. The reaction solution was prepared to contain a final concentration of 0.15 mM DPPH and 0.8 mM Tris hydrochloride (Promega, Wisconsin, USA) in 52 % EtOH and incubated for 20 min in the dark. Then, the absorbance of the reaction solution was measured at 520 nm. Trolox (Wako Pure Chemical Industrial Co., Ltd) was used as the standard. In addition, the RSA of bilirubin, urobilinogen, stercobilin, and Ge-132 was investigated by performing a DPPH assay and compared with the antioxidant capacity of vitamin C.

### 2.10 Analysis of the ratio of glutathione in erythrocytes

Frozen RBCs were added to 5 % 5-sulfosalicylic acid dihydrate (SSA) (Wako Pure Chemical Industrial Co., Ltd.) for lysis. The cell lysis solution was centrifuged at 8000×*g* for 10 min at 4 °C. The supernatant was transferred to a new tube, and deionized water was added to reduce the SSA concentration to 0.5 % for the assay. The amounts of glutathione (GSH) and glutathione disulfide (GSSG) in RBCs were measured using a GSSG/GSH Quantification Kit (Dojindo Molecular Technologies, Inc., Japan) according to the manufacturer's instructions.

### 2.11 Counting of the colonies of burst-forming unit-erythroid (BFU-E) cells formed in bone marrow cells (BMCs)

Seven-week-old male ICR mice were housed under the same conditions as in the other experiments. During acclimation, all animals were provided a chow diet. After the acclimation period, the mice were assigned randomly to three groups: a group fed a chow diet, a group fed a 0.05 % Ge-132 diet for 4 days, and a group fed a 0.05 % Ge-132 diet for 7 days. Ten mice were included in each group. After the mice were fed each experimental diet, we dissected the animals under anesthesia and collected the left femurs in a sterile environment. Excess flesh and skin were removed from the femurs. The upper and lower parts of the bone were excised in a Petri dish. According to the protocol recommended by Veritas Corporation, the BMCs were collected by pouring PBS into the bone. An equal amount of Iscove's modified Dulbecco's medium (Thermo Fischer Scientific, Massachusetts, USA) containing 2 % FBS was added to the collected cell suspension. The number of cells was counted and adjusted to 2.5 × 10^6^ cells/ml. The cell suspension was diluted 10-fold with MethoCult medium M3334 (Veritas Corporation, Tokyo, Japan) and mixed thoroughly. One aliquot of medium containing suspended cells was added to a 35 mm Petri dish, and the medium was extended uniformly throughout the Petri dish. The prepared Petri dish was cultured at 37 °C with 5 % CO_2_ for two weeks, and then the erythroblast colonies were counted with an inverted microscope.

### 2.12 Statistical analysis

The results are presented as the means ± standard deviations (SDs). Statistical analyses were performed using Student's *t*-test or Dunnett's and Steel's methods for multiple comparisons. For the analyses of fecal amounts of erythrocyte-metabolizing pigments and antioxidant activity, the value obtained on day 0 of the experimental diet for each group was used as a control. The statistical significance of all experiments was defined as p < 0.05 or p < 0.01.

## 3 Results

### 3.1 Effect of THGP on the phagocytic activity of RAW264.7 cells

**Fig. 1:**
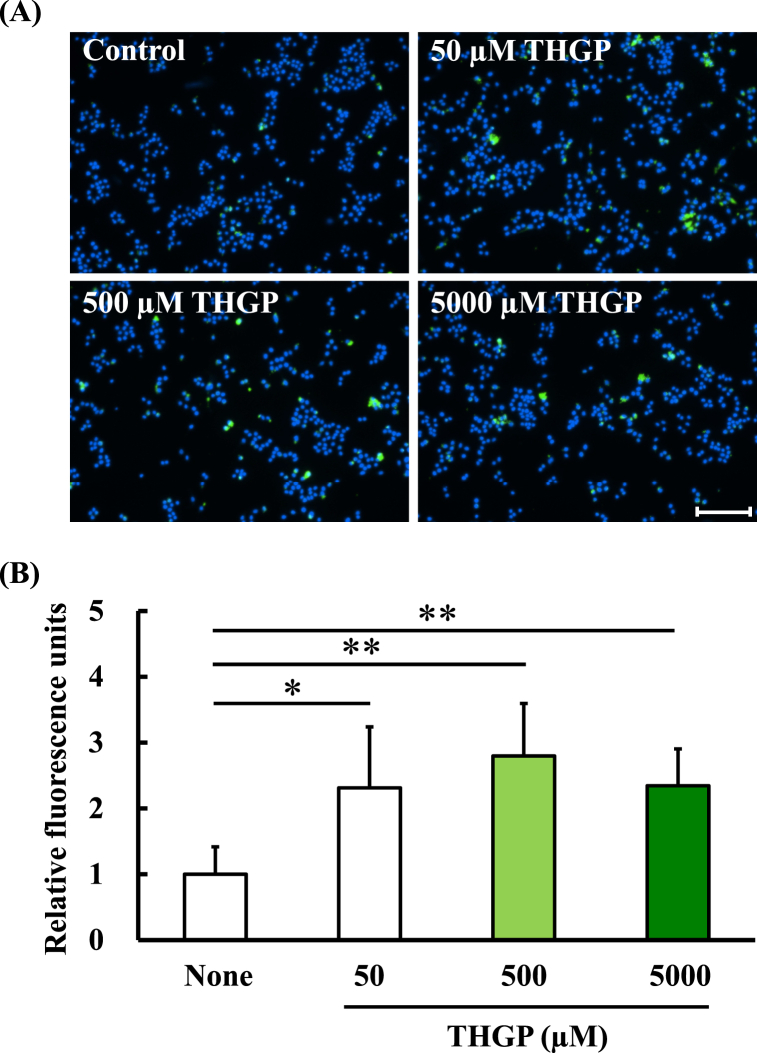
Evaluation of phagocytosis in RAW264.7 cells treated with THGP. (A) Fluorescence photographs showing a phagocytosis assay conducted using RAW264.7 cells treated or not treated with THGP for 1 day. Green and blue colors in the photographs represent the phagocytosed FITC-labeled beads and the cell nucleus (Hoechst 33452), respectively. (B) The ratio of FITC-labeled beads phagocytosed by the THGP-treated group to that in the control group was analyzed. The data are presented as the means, and the bars indicate the SDs (n = 6). The asterisk and double asterisk indicate significant differences at p < 0.05 and 0.01, respectively. The scale bar represents 100 μm.

We first examined the effect of THGP on the phagocytic activity of RAW264.7 cells, a murine macrophage line. After one day of treatment with THGP, FITC-labeled beads were added to the culture of RAW264.7 cells and incubated for 1 h. Photographs showed that the number of FITC beads was clearly greater for THGP-treated RAW264.7 cells than for untreated control cells (Fig. 1A). The results were calculated as the ratio of phagocytosed FITC-labeled beads in the THGP-treated groups to those in the control group (Fig. 1B). The fluorescence values obtained with 0, 50, 500 and 5000 μM THGP addition were 1.0 ± 0.4, 2.3 ± 0.9, 2.8 ± 0.8 and 2.3 ± 0.6 units, respectively. Compared with that of the control group, FITC-labeled bead uptake by RAW264.7 cells was significantly increased 2- and 3-fold after treatment with THGP. The addition of 500 μM THGP resulted in the highest uptake of fluorescent beads; thus, 500 μM THGP was used for the next erythrocyte phagocytosis test.

### 3.2 Effects of THGP on erythrocyte phagocytosis-related functions of RAW264.7 cells

**Fig. 2:**
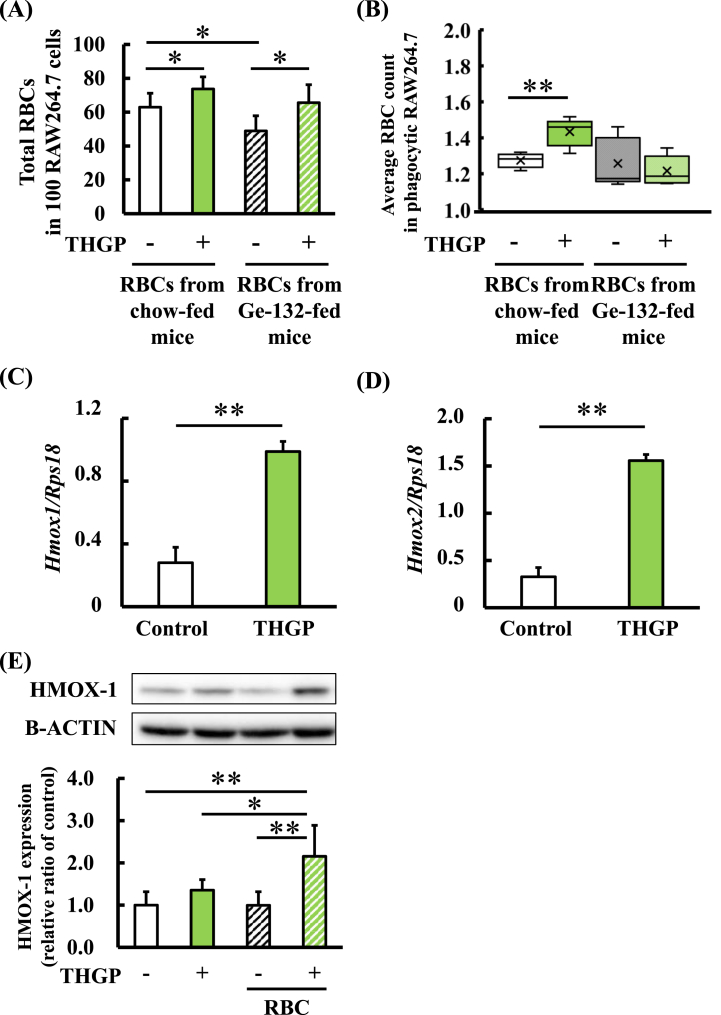
Effect of THGP on erythrocyte phagocytosis and heme oxygenase gene and protein expression in RAW264.7 cells. The effect of 500 μM THGP on the ability of RAW264.7 cells to phagocytose erythrocytes was evaluated by optical microscopy. (A) The number of phagocytosed RBCs per 100 macrophages was calculated by dividing the number of phagocytosed erythrocytes by the number of RAW264.7 cells and correcting to 100. (B) The average number of phagocytosed RBCs in phagocytic macrophages was calculated by dividing the number of phagocytosed RBCs by the number of phagocytic RAW264.7 cells. Total RNA was extracted from RAW264.7 cells treated or not treated with 500 μM THGP. The expression levels of the Hmox1 (C) and Hmox2 (D) mRNAs were measured by RT–qPCR and normalized to the expression of the RPS18 reference gene. Protein was extracted from RAW264.7 cells treated or not treated with 500 μM THGP and erythrocytes. (E) The expression levels of the HMOX-1 protein were measured by western blotting and normalized to the expression of β-actin. Non-adjusted images of Western blotting analysis were presented in Supplemental Fig. 5. The data are presented as the means, and the bars indicate the SDs (n = 5–6). The asterisks and double asterisks indicate significant differences at p < 0.05 and 0.01, respectively.

We next investigated the effect of THGP on erythrocyte phagocytosis by macrophages. RBCs were obtained from normal chow-fed mice (RBCs in chow-fed mice) and Ge-132-fed mice (RBCs in Ge-132-fed mice). RAW264.7 cells were treated with or without 500 μM THGP for 1 day, and RBCs from each group were added to the culture. Fig. 2A shows the number of erythrocytes phagocytosed per 100 RAW264.7 cells. THGP significantly increased the total number of erythrocytes phagocytosed per 100 RAW264.7 cells compared with that of the control group. Briefly, the total number of erythrocytes phagocytosed by RAW264.7 cells treated or not treated with THGP was 74 ± 7 cells or 63 ± 8 cells using RBCs from chow diet-fed mice and 66 ± 11 cells or 49 ± 9 cells using RBCs from Ge-132 diet-fed mice, respectively. Fig. 2B shows the average number of erythrocytes phagocytosed by phagocytic RAW264.7 cells. The average number of erythrocytes phagocytosed by phagocytic RAW264.7 cells was significantly increased after THGP treatment using RBCs from chow diet-fed mice, but an increase was not observed using RBCs from Ge-132 diet-fed mice. Briefly, the average number of erythrocytes phagocytosed by phagocytic RAW264.7 cells treated or not treated with THGP was 1.4 ± 0.08 cells or 1.3 ± 0.04 cells, respectively, using RBCs from chow diet-fed mice. The average number of erythrocytes phagocytosed by phagocytic RAW264.7 cells treated or not treated with THGP was 1.2 ± 0.08 cells or 1.3 ± 0.14 cells, respectively, using RBCs from Ge-132 diet-fed mice. To explain why THGP treatment was not effective in RBCs from Ge-132 diet-fed mice, as shown in Fig. 2B, the ratio of phagocytic macrophages was calculated and is shown in Supplemental Fig. 1(S1). Supplemental Table S1 shows the values used to calculate the values in Fig. 2A and B and Fig. S1. The values in Fig. S1 were calculated by dividing the number of phagocytic RAW264.7 cells by the number of RAW264.7 cells. As shown in Fig. S1, the treatment of macrophages with THGP significantly increased the ratio of phagocytic RAW264.7 cells among the RBCs of Ge-132 diet-fed mice. Therefore, we reasoned that when erythrocytes from Ge-132-fed mice were used, THGP treatment may not have changed the average number of phagocytosed RBCs due to an increased proportion of phagocytic macrophages. On the other hand, THGP treatment activated macrophages and increased the total number of phagocytosed RBCs (Fig. 2A). This increase was likely caused by an increase in the average number of erythrocytes phagocytosed by phagocytic RAW264.7 cells (Fig. 2B) or the ratio of phagocytic macrophages (Fig. S1). Therefore, THGP may reduce the number of aged RBCs by inducing macrophage-mediated phagocytosis. Furthermore, we investigated the expression of the genes encoding the heme oxygenase enzyme that metabolizes heme produced by RBC degradation. Heme oxygenase is a rate-determining enzyme involved in heme metabolism. Fig. 2C and D shows heme oxygenase mRNA expression in RAW264.7 cells treated or not treated with 500 μM THGP. The mRNA expression levels of Hmox1 and Hmox2 were significantly increased upon THGP addition. Briefly, Hmox1 gene expression was 0.99 ± 0.31 or 0.28 ± 0.09, and Hmox2 gene expression was 1.56 ± 0.41 or 0.33 ± 0.21, with or without THGP treatment, respectively. Furthermore, the level of HMOX-1 was slightly increased following 500 μM THGP addition and significantly increased after treatment with both 500 μM THGP and RBCs (Fig. 2E). Briefly, HMOX-1 protein expression was 1.35 ± 0.25 or 1.00 ± 0.32 with or without THGP treatment and 2.15 ± 0.73 or 1.00 ± 0.32 with or without THGP treatment and with the addition of RBCs, respectively. Therefore, THGP increases RBC degradation by activating macrophages.

### 3.3 Quantitative evaluation of erythrocyte pigment metabolism and RSA in murine feces after Ge-132 intake

**Fig. 3:**
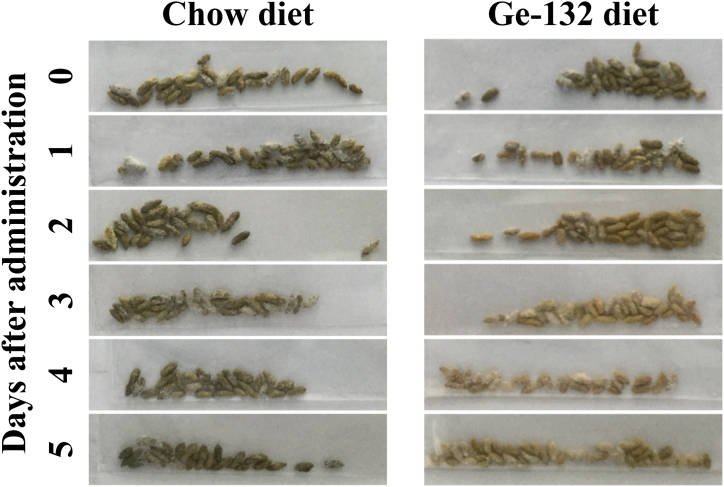
Effect of Ge-132 ingestion on the color of mouse feces. After a three-week acclimation period, the mice were randomly assigned to the chow diet group and a 0.05 % Ge-132 diet group. Fecal samples were collected when animals began feeding on each experimental diet. The photographs show the colors of feces from mice fed the chow diet and the Ge-132 diet.

THGP enhanced erythrocyte phagocytosis by macrophages (Fig. 2A). These data suggested that the ingestion of Ge-132 may enhance RBC degradation. Most of the metabolized pigments of RBCs are excreted in feces. According to these findings, we next collected feces from mice fed a diet supplemented with or without Ge-132 and observed the fecal color to investigate whether Ge-132 ingestion promoted the decomposition of erythrocytes. Fig. 3 shows the collected feces. The color of the feces did not change in the chow diet group; however, the color in the 0.05 % Ge-132 group turned bright yellow on the third experimental day.

**Fig. 4:**
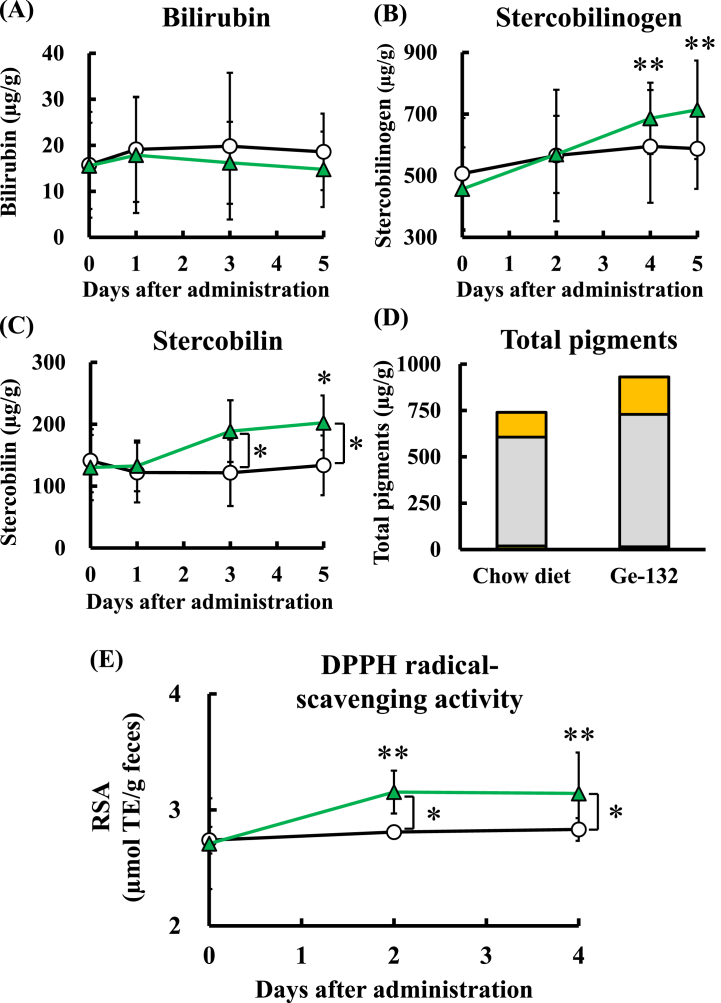
Evaluation of the contents of pigments derived from RBC metabolism and RSA in feces following Ge-132 ingestion. Fecal samples were collected from mice fed a 0.05 % Ge-132 diet or control diet. The amounts of bilirubin (A) and stercobilin (B) in feces were measured using HPLC, and the content of stercobilinogen (C) in feces was measured by a colorimetric method. Circles and triangles represent the chow diet group and 0.05 % Ge-132 diet group, respectively. (D) Yellow, gray or orange bars in the total pigment graphs show the contents of bilirubin, stercobilinogen or stercobilin, respectively. (E) The extent of the change in fecal RSA over time was measured. The data are presented as the means, and the bars indicate the SDs (n = 7–8). The asterisk and double asterisk indicate significant differences at p < 0.05 and 0.01, respectively. In the analyses of the fecal amounts of erythrocyte-metabolizing pigments and antioxidant activity, the value obtained on day 0 of the administration of the experimental diet was used as a control for each group. The brackets indicate significant differences at p < 0.05 compared with the chow diet group.

Since the change in fecal color was clarified to be caused by Ge-132 intake, the amounts of the metabolized pigments bilirubin, stercobilinogen and stercobilin derived from erythrocytes were measured in feces. A significant increase in the amount of bilirubin was not observed after Ge-132 ingestion (Fig. 4A). The amount of stercobilinogen was significantly greater on day 4 (686 ± 116 μg/g) and day 5 (714 ± 160 μg/g) of Ge-132 ingestion than on day 0 (457 ± 135 μg/g) (Fig. 4B). Furthermore, a significant increase in the amount of stercobilin was confirmed on day 5 (202 ± 48 μg/g) of Ge-132 ingestion compared to day 0 (130 ± 51 μg/g) (Fig. 4C). Bilirubin is a pigment produced by heme degradation and is excreted from the liver into the intestinal tract via bile. Furthermore, bilirubin is reduced by intestinal bacteria and converted to urobilinogen. Then, part of it is reabsorbed, but most is excreted in feces as stercobilinogen or stercobilin [28,34]. Therefore, bilirubin did not remain, and its levels did not increase in the feces after Ge-132 ingestion (Fig. 4D). Additionally, when the color of the feces began to change on the third day of Ge-132 intake, the amount of stercobilin (which gives feces a yellow‒brown color) on day 3 of Ge-132 ingestion (189 ± 54 μg/g) was larger than that on day 3 of consumption of the chow diet (122 ± 50 μg/g). These data suggested that the change in the fecal color after the ingestion of Ge-132 was due to increases in the amounts of pigments produced during metabolism in erythrocytes.

The amount of stercobilinogen was increased in the feces of mice fed Ge-132. Stercobilinogen is a structural isomer of urobilinogen, and urobilinogen has been reported to have high RSA [35]. We investigated the antioxidant capacity in feces accompanying the increase in pigment levels by measuring the DPPH RSA. The RSA of feces is shown in Fig. 4E and is reported in Trolox equivalents (TEs). The RSA was significantly greater on days 2 and 4 of Ge-132 ingestion (3.15 ± 0.18 and 3.14 ± 0.35 μmol TE/g) than in the chow diet-fed group (2.81 ± 0.05 and 2.83 ± 0.10 μmol TE/g) or on day 0 (2.71 ± 0.39 μmol TE/g). However, THGP itself had no RSA (Supplemental Table 2). The antioxidant properties of feces increased with the increase in the fecal levels of pigments derived from RBC metabolism.

### 3.4 Effect of Ge-132 on the hematocrit value, ratio of GSH to GSSG and BMC colony formation

**Fig. 5:**
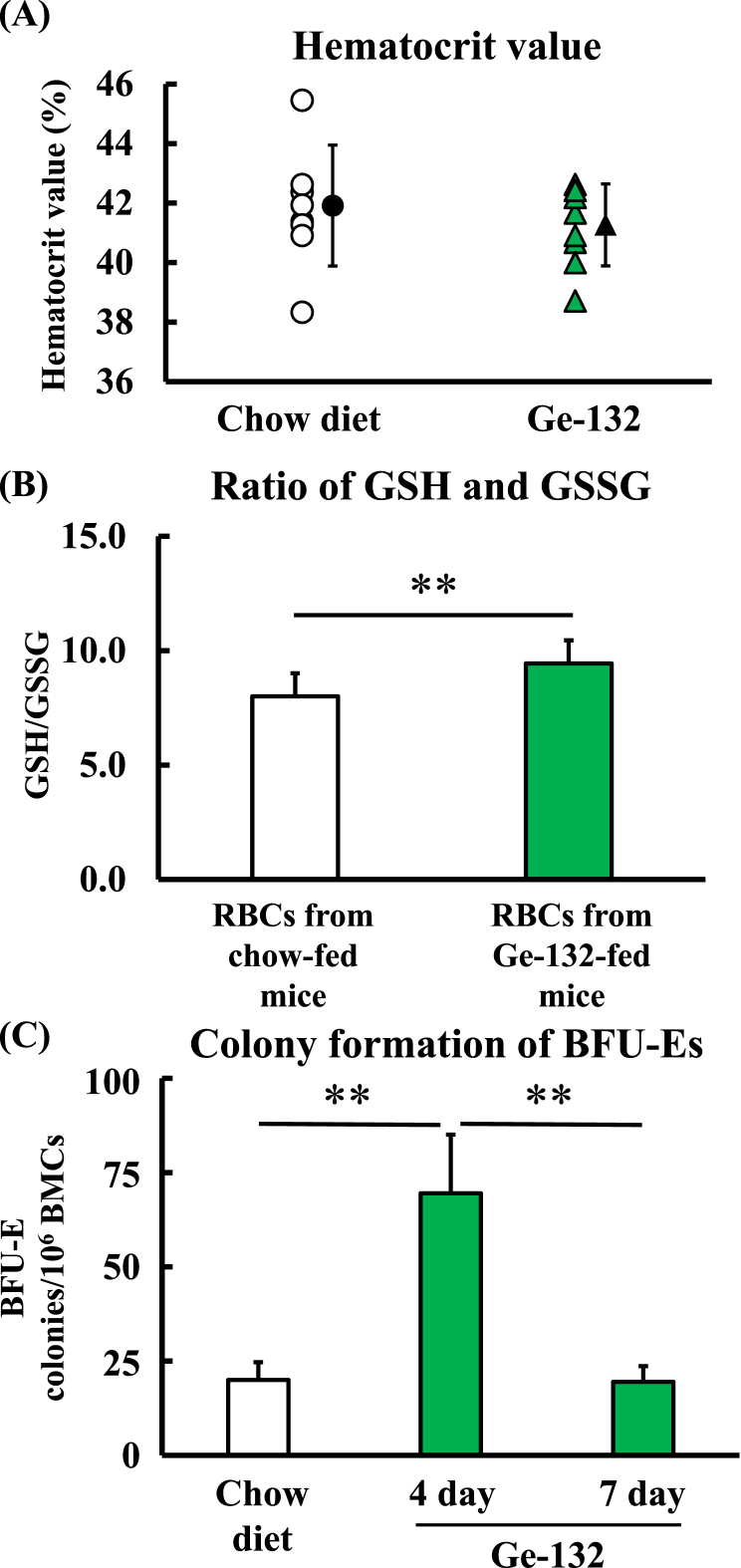
Effect of Ge-132 ingestion on hematocrit value and the ratio of GSH and GSSG in erythrocytes and colony formation of BFU-Es in BMCs. The hematocrit value of mice fed a diet with or without 0.05 % Ge-132 was measured (A). The data are presented as the means, and the bars indicate the SDs (n = 8). The amounts of GSH and GSSG were measured using commercial kits (B). The data are presented as the means, and the bars indicate the SDs (n = 6). BMCs were obtained from mice fed a 0.05 % Ge-132 diet or control diet. BMCs were collected and then cultured in MethoCult medium M3334. After 2 weeks of culture, the erythroblast colonies were counted (C). The data are presented as the means, and the bars indicate the SDs (n = 8–10). The double asterisk indicates significant differences at p < 0.01.

Because the ingestion of Ge-132 promoted RBC degradation, we investigated the hematocrit value, assessing the percentage of RBCs in the blood. As the hematocrit value was 41.8 ± 2.0 % in chow diet-fed mice and 41.1 ± 1.3 % in Ge-132 diet-fed mice, the hematocrit level was not reduced by the ingestion of Ge-132 (Fig. 5A). Erythrocyte aging decreases the levels of GSH-producing enzymes and glutathione reductase, and aged RBCs exhibit decreased GSH levels and increased GSSG levels [36]. Therefore, we examined the effect of Ge-132 on the redox ratio of glutathione in erythrocytes. The ratio of GSH to GSSG was 8.0 ± 1.0 in RBCs from chow-fed mice but significantly increased to 9.4 ± 1.0 in erythrocytes from mice in the 0.05 % Ge-132 diet-fed group (Fig. 5B). Finally, we evaluated the effect of Ge-132 ingestion on hematopoiesis by measuring BFU-E colonization of BMCs (Fig. 5C). BMC colony formation measured as BFU-Es was 20.0 ± 4.7 and 69.5 ± 15.6 in mice fed the chow diet and 0.05 % Ge-132 diet for 4 days, respectively. The colonies of BFU-Es in Ge-132 diet-fed mice were significantly increased by approximately 3.5-fold compared to that of animals fed the chow diet. However, BMC colony formation returned to 19.5 ± 4.2 after 7 days.

## 4 Discussion

RBCs are cells that specialize in transporting oxygen; thus, their cell membranes become oxidized as they repeatedly take in and let out oxygen, reducing their inherent extremely high deformability. Senescent RBCs, which exhibit reduced deformability, are known to be prone to agglutination [37]. The increased likelihood of aggregation of senescent RBCs results in reduced blood flow and oxygen-carrying capacity. A decreased oxygen-carrying capacity and impaired blood flow hinder the supply of oxygen to the whole body and are considered to interfere with the activity of cells throughout the body. Elimination of senescent RBCs is therefore important to maintain proper body function.

In this study, we clarified that the addition of THGP, a monomeric hydrolyzed molecule of Ge-132, enhanced the ability of macrophages to phagocytize erythrocytes through upregulation of HMOX-1 (Fig. 2). HMOX-1 has been reported to indirectly enhance macrophage-mediated phagocytosis [38]. Since an increase in the intracellular heme content reduces phagocytosis, HMOX-1activation promotes phagocytosis through rapid heme degradation. Therefore, it is speculated that THGP treatment promotes heme metabolism and RBC phagocytosis in macrophages, which enables the rapid disposal of unwanted senescent erythrocytes. We found that Ge-132 intake increased the levels of erythrocyte-degrading pigments in feces (Fig. 4). We also showed that the GSH/GSSG ratio in erythrocytes, which is an erythrocyte aging marker [36], was decreased after the ingestion of Ge-132 (Fig. 5B). These data may support the notion that Ge-132 promotes the degradation of senescent RBCs. However, the GSH/GSSG ratio can be influenced by various environmental factors and is insufficient as a marker of the aging RBC proportion. Therefore, future studies will measure other erythrocyte senescence markers and evaluate the reduction in the erythrocyte senescence proportion after Ge-132 intake using different approaches. In addition, fewer erythrocytes from Ge-132-fed mice than erythrocytes from control mice were phagocytosed by RAW264.7 cells (Fig. 2A). In other words, macrophages in Ge-132-fed mice have a greater ability to find and phagocytize aged erythrocytes than those in chow diet-fed mice, suggesting that aged erythrocytes are sufficiently phagocytosed in the mouse body (Supplemental Fig. 2). In brief, the results suggest that the ingestion of Ge-132 promotes the degradation of senescent erythrocytes even *in vivo* by activating macrophage-mediated phagocytosis. However, whether the activation of the erythrocyte phagocytic ability of macrophages by Ge-132 observed *in vitro* actually occurs *in vivo* is a topic for future research.

Although Ge-132 intake accelerated erythrocyte decomposition, it did not affect the hematocrit value (Fig. 5A). In general, RBC removal and erythropoiesis are balanced in the homeostatic situation [6]. When anemia or hypoxia occurs in which the number of RBCs decreases, the production of RBCs is promoted [39]. These findings suggest that when Ge-132 ingestion increased erythrocyte degradation, erythrocyte production was promoted to maintain homeostasis. An investigation of the effect of Ge-132 on erythropoiesis revealed that ingestion of Ge-132 for 4 days enhanced the production of erythroblasts in mouse bone marrow cells (Fig. 5C) and that the production returned to the control levels after 7 days of Ge-132 ingestion. These data showed that the number of senescent RBCs that needed to be removed was decreased after 7 days of Ge-132 intake, and during this time, decomposition may have stabilized, and the production amount may have decreased. Therefore, an increase in production for approximately 4–5 days was sufficient to compensate for the amount of decomposition. Furthermore, the reticulocyte percentage is an indicator of RBC production. Supplemental Fig. 3 shows the ratio of reticulocytes five days after ingestion of Ge-132. ingestion of Ge-132 also increased the number of reticulocytes, although this increase was not significant. Briefly, the ratio of reticulocytes in chow diet-fed or Ge-132 diet-fed mice was 1.0 ± 0.8 % or 1.8 ± 0.6 %, respectively. In addition, the increase in erythrocyte metabolic pigments shown in Fig. 4B and C stabilized after 4–5 days, suggesting that the decomposition of aged erythrocytes had stabilized. Therefore, we hypothesized that erythrocytes had already matured by the 7th day after the initiation of Ge-132 ingestion, whereas the number of immature erythrocytes was still increasing on day 4. Thus, the findings suggested that to compensate for the increased amount of erythrocyte decomposition caused by Ge-132 intake, the production of erythrocytes may be promoted, and the number of erythrocytes may be maintained *in vivo*. However, it remains unclear whether the promotion of RBC metabolism by ingesting Ge-132 affects the lifespan of RBCs. This question will be a topic for future research. Regarding hematopoietic factors, Ge-132 intake did not increase the amount of erythropoietin, which acts on bone marrow hematopoietic stem cells to promote erythroid differentiation (Supplemental Fig. 4). Therefore, the promotion of erythropoiesis through the maintenance of homeostasis by Ge-132 intake may be another erythropoiesis mechanism not mediated by erythropoietin. Macrophages also regulate erythropoiesis [40,41]. Macrophages phagocytize aged RBCs, and HMOX-1 cleaves the porphyrin ring of the cyclic compound heme to produce iron, biliverdin, and carbon monoxide [4,42,43]. Released iron is stored as ferritin and reused for RBC regeneration in the bone marrow. Ferritin produced by macrophages is endocytosed into erythroblasts and utilized for heme production in erythroid progenitor cells [40]. Furthermore, it has been previously reported that macrophages play a functional role not only in supplying iron but also in promoting erythropoiesis by directly stimulating the proliferation of erythroblasts [40,41,44]. Therefore, it is future research to clarify whether the promotion of erythroblast differentiation is due to macrophage activation by Ge-132.

These findings suggest that ingestion of Ge-132 activates macrophages and promotes the degradation of aged RBCs in the reticuloendothelial system and the formation of new RBCs in the bone marrow. Antioxidant substances such as vitamin E and polyphenols have been reported to alleviate oxidative damage to RBC membranes [45,46]. Both molecules reduce oxidative damage to erythrocyte membranes and inhibit erythrocyte senescence. In this study, the ingestion of Ge-132 activated macrophages and rapidly eliminated senescent RBCs, and this treatment is expected to ameliorate the blood flow disturbance and oxygen carrying capacity reduction that may occur due to an increase in the number of senescent RBCs. The effect of Ge-132 on hypertension, which is actually a blood flow disorder, has been reported [47]. However, the mechanism has not yet been elucidated. The elimination of senescent RBCs by Ge-132 promotes blood flow improvement, which may represent one of the blood pressure-lowering mechanisms of Ge-132. In future research, it will be desirable to investigate whether ingestion of Ge-132 improves blood flow by rapidly removing aged RBCs.

Ingestion of Ge-132 reportedly increases blood oxygen levels at high altitudes and suppresses increases in the respiratory rate [48]. One speculated reason is that Ge-132 ingestion improves oxygen transport throughout the body due to the removal of senescent RBCs with weakened function. Changes in systemic oxygen carrying capacity are associated with muscle performance and fatigue [49]. An insufficient oxygen supply to peripheral tissues induces fatigue by promoting the accumulation of metabolic byproducts, which impedes excitation and contraction within muscle cells. The improvement of oxygen transport in the body after Ge-132 intake is thought to increase the supply of oxygen to each cell, increase energy production, and enhance cell function. As a result, individual organs composed of each cell type function normally, which may improve exercise performance and promote recovery from fatigue.

Finally, the usefulness of the substances produced during heme decomposition is worth mentioning. Ge-132 intake increased the fecal level of stercobilinogen, a precursor of stercobilin. Stercobilinogen is a structural isomer of urobilinogen. Urobilinogen is a low-molecular-weight antioxidant *in vivo* and has been reported to have higher antioxidant activity than vitamin E [35]. Furthermore, stercobilin was shown to exhibit antioxidant properties equivalent to those of other general antioxidants (Supplemental Table 2). As shown by these data and those in Fig. 4B and C, the increase in the antioxidant capacity of both urobilinoids in feces on day 5 of Ge-132 ingestion compared to day 0 was 860 nmol TE/day for stercobilinogen and 106 nmol TE/day for stercobilin. Since the authors have already confirmed that Ge-132 itself has no antioxidant activity [21], the increases in fecal stercobilinogen and stercobilin contents due to Ge-132 ingestion may mediate the antioxidant effect of Ge-132. The antioxidant capacity in feces also indicates the antioxidant capacity of the colonic lumen [50]. Thus, the induction of antioxidant pigment production by Ge-132 may mitigate oxidative stress-induced damage in the colonic lumen. Inflammatory bowel disease (IBD), which manifests as ulcerative colitis or Crohn's disease, is a chronic gastrointestinal disease, and oxidative stress has been shown to play an important role in IBD pathogenesis and progression [51]. Vitamin C, which has antioxidant properties, has been reported to ameliorate inflammation associated with ulcerative colitis and early colon cancer [52,53]. One of the findings from this study is that stercobilinogen in the gastrointestinal tract is a molecule responsible for scavenging radicals in the large intestine. Weight conversion from the recommended daily vitamin C intake of 90 mg for males [54] yields an estimated RSA of only 0.25 nmol TE/day equivalent in mice. As mentioned above, the RSA of stercobilinogen, the levels of which increased on the fifth day of Ge-132 intake, was surprisingly 860 nmol TE/day, which is approximately 3000 times higher than the value of 0.25 nmol TE/day for vitamin C. Considering these findings, the induction of antioxidant production by Ge-132 may be more useful in ameliorating IBD than vitamin C intake.

In summary, the findings suggest that ingestion of Ge-132 activates senescent erythrocyte degradation in macrophages and increases erythrocyte production to compensate for the decreased erythrocyte mass. However, this experiment used healthy mice and established cell lines. Therefore, a future task is to evaluate whether the reduction in the number of senescent RBCs mediated by Ge-132 intake improves health status *in vivo*.

## 5 Conclusions

We found that THGP-treated macrophages exhibited enhanced phagocytosis of foreign substances and erythrocytes. Furthermore, in mice, ingestion of Ge-132 enhanced erythrocyte decomposition and increased the fecal levels of pigments derived from heme metabolism, such as stercobilinogen and stercobilin. Furthermore, these pigments were shown to possess antioxidant activity and increase the antioxidant capacity in the intestinal tract. It has been revealed that ingestion of Ge-132 promotes not only the decomposition of erythrocytes but also erythroblast differentiation in bone marrow cells by resulting in homeostasis between the supply and removal of RBCs.

## Ethics statement

The animal experiments were all conducted at Asai Germanium Research Institute Co., Ltd., according to the guidelines provided by the Ethics Committee of Experimental Care, which were based on public guidelines established by the Japanese Ministry of Education, Culture, Sports, Science and Technology. The studies were individually approved according to the ethical guidelines of the judging committee at Asai Germanium Research Institute Co., Ltd. (approval number: 2010-01; date of approval: 2/16/2010, approval number: 2015-01; date of approval: October 3, 2015, approval number: 2019-01; date of approval: April 6, 2019).

## Funding

This research received funding from Asai Germanium Research Institute Co., Ltd.

## Data availability statement

Raw data were generated at Asai Germanium Research Institute Co., Ltd. The data related to the study are not stored in a public repository. However, derived data supporting the findings of this study are available from the corresponding author T.T. on request.

## CRediT authorship contribution statement

**Tomoya Takeda:** Writing – review & editing, Writing – original draft, Visualization, Project administration, Investigation, Formal analysis, Data curation, Conceptualization. **Junya Azumi:** Writing – review & editing, Investigation. **Mika Masaki:** Writing – review & editing, Investigation. **Takae Nagasawa:** Writing – review & editing, Investigation. **Yasuhiro Shimada:** Writing – review & editing, Investigation. **Hisashi Aso:** Writing – review & editing, Supervision. **Takashi Nakamura:** Writing – review & editing, Supervision, Project administration, Investigation, Conceptualization.

## Declaration of competing interest

Takashi Nakamura received remuneration from Asai Germanium Research Institute Co., Ltd., as an officer. Hisashi Aso received remuneration from Asai Germanium Research Institute Co., Ltd., as an adviser. Others are employed by Asai Germanium Research Institute, Co., Ltd.
