# Supplementary material for: Organogermanium, Ge-132, promotes the clearance of senescent red blood cells via macrophage-mediated phagocyte activation
Source: Heliyon. 2023 Dec 3;10(1):e23296. doi: 10.1016/j.heliyon.2023.e23296 (PMC10754881; doi:10.1016/j.heliyon.2023.e23296)
Supplement: Multimedia component 1 [file mmc1.docx]

**Organogermanium, Ge-132, promotes the clearance of senescent red blood cells via macrophage-mediated phagocyte activation**

Tomoya Takeda^a*^, Junya Azumi^a^, Mika Masaki^a^, Takae Nagasawa^a^, Yasuhiro Shimada^a^, Hisashi Aso^b^, Takashi Nakamura^a^

^a^Asai Germanium Research Institute Co., Ltd., 3-131, Suzuranoka, Hakodate, Hokkaido 042-0958, Japan

^b^Laboratory of Animal Health Science, Graduate School of Agricultural Science, Tohoku University, 468-1 Aramaki aza, Aoba, Sendai, Miyagi 980-8578 Japan

***** Corresponding author at: Asai Germanium Research Institute Co., Ltd., 3-131, Suzuranoka, Hakodate, Hokkaido 042-0958, Japan
E-mail address: tomo.t621@asai-ge.co.jp (Tomoya Takeda)

**Supplementary Material and Methods**

## Analysis of the ratio of phagocytic macrophages

## RAW264.7 cells were prepared using the method described in section 2.3, and red blood cell (RBC) phagocytosis by macrophages was observed. The percentage was calculated by dividing the number of phagocytic RAW264.7 cells by the number of RAW264.7 cells. The values shown in Table S1 were used in the calculation.

## Analysis of the percentage of reticulocytes in erythrocytes

## The blood of experimental mice whose fecal color was observed was used. After being fed the experimental diet for five days, the animals were dissected, and whole blood was collected. The collected blood was mixed with 1% brilliant cresyl blue solution (Muto Pure Chemicals Co., Ltd.). One drop of the mixed solution was dropped on a slide glass, and the solution was spread thinly with a cover glass. After drying, the surface coated with the mixed solution was photographed with a BX40 biological microscope (Olympus Co., Tokyo, Japan) and a Moticam 2000 (Shimadzu Co., Kyoto, Japan) using an oil immersion lens. Ten fields of each sample were observed, and the stained reticulocytes and RBCs were counted.

## Analysis of the concentration of plasma erythropoietin

## The blood of experimental mice was used to measure the ability of RAW264.7 cells to phagocytize RBCs. After being fed the experimental diet for four days, the animals were dissected, and whole blood was collected. The collected blood was centrifuged at 850 × g for 10 min. The supernatant consisting of the blood plasma was used. The amount of erythropoietin in the supernatant was measured using a Mouse Erythropoietin ELISA Kit (Proteintech Group, Inc., Rosemont, IL, USA) according to the manufacturer’s instructions.

# Supplementary Data

**Figure S1 Effect of Ge-132 ingestion on the percentage of phagocytic macrophages**

The percentage of phagocytic RAW264.7 cells was quantified. The values shown in Table S1 were used in the calculation. The percentage was calculated by dividing the number of phagocytic RAW264.7 cells by the number of RAW264.7 cells. The data are presented as the means, and the bars indicate the SDs (n = 6). The asterisk indicates significant differences at p < 0.05.

*

**% of phagocytic RAW264.7 in RAW264.7**

**THGP**

**RBCs from chow-fed mice**

**RBCs from Ge-132-fed mice**

**Figure S2 Scheme of the mechanism through which Ge-132 intake increases macrophage-mediated phagocytosis and reduces the number of senescent RBCs.**

After the seeding of erythrocytes on RAW264.7 cells, the count of phagocytosed senescent erythrocytes in Ge-132-fed mice is lower than that in chew-fed mice. The ingestion of Ge-132 may increase erythrocyte phagocytosis by macrophages and consequently decrease the number of senescent erythrocytes in the blood.


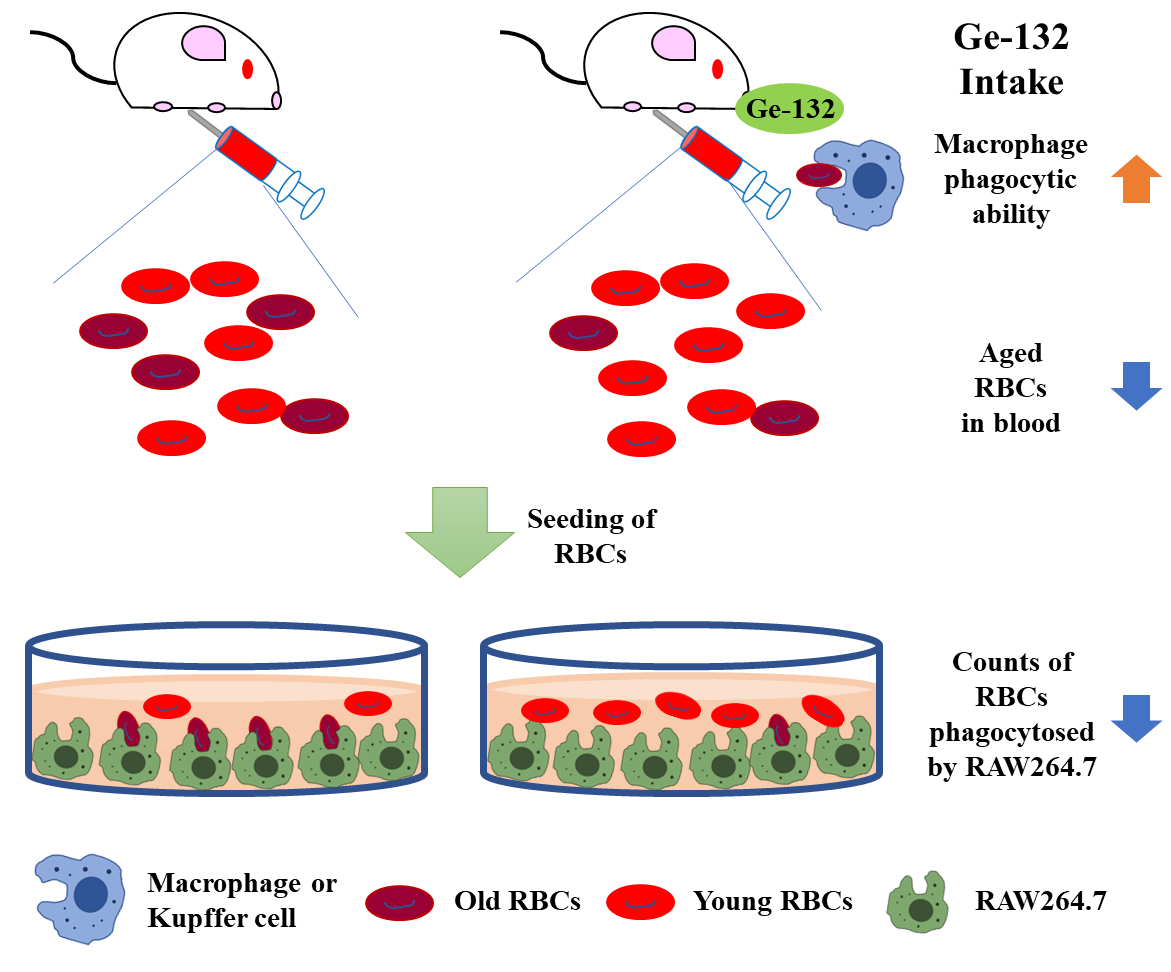


**Figure S3 Effect of Ge-132 ingestion on the numbers of reticulocytes in red blood cells.**

The reticulocytes were counted in the RBCs of mice fed a diet with or without 0.05% Ge-132 for 5 days. (A) The photograph shows a typical reticulocyte in the RBCs. The arrow indicates a reticulocyte with fragments of nucleaus. (B) The percentage of reticulocytes per RBC for each sample is shown. The data are presented as the means, and the bars indicate the SDs (n = 8).

**(A)**


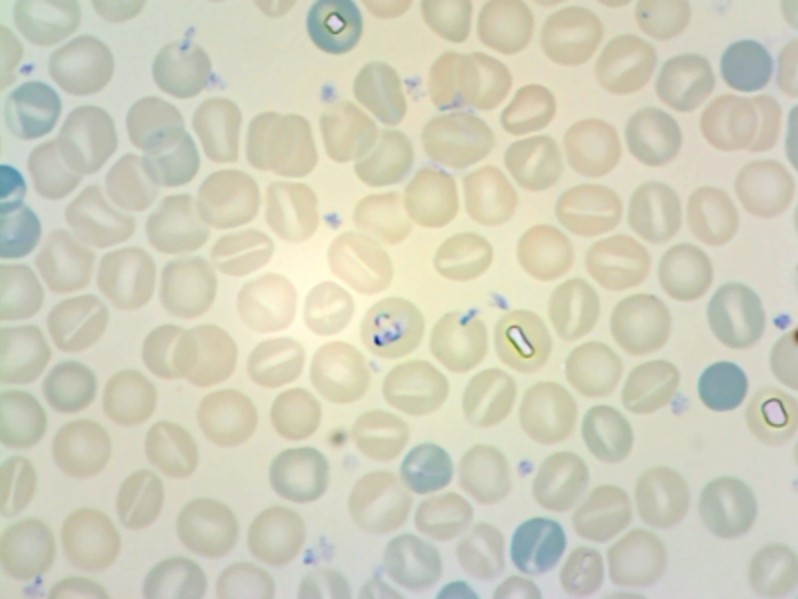


**(B)**

**p=0.060**

**% of reticulocytes in RBCs**

**Figure S4 Effect of Ge-132 ingestion on the concentration of plasma erythropoietin.**

The concentration of erythropoietin was measured in the plasma of mice fed a diet with or without 0.05% Ge-132. The data are presented as the means, and the bars indicate the SDs (n = 6).

**Erythropoietin (pmol/ml)**

**Figure S5 Effect of THGP on heme oxygenase protein expression in RAW264.7 cells.**

The original uncropped gel image was shown in Supplementary Figure 5.

Exposure time: 1sec


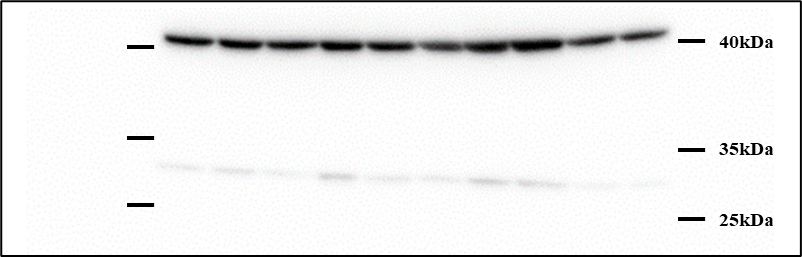


β-ACTIN

Used in the manuscript

Exposure time: 10 sec


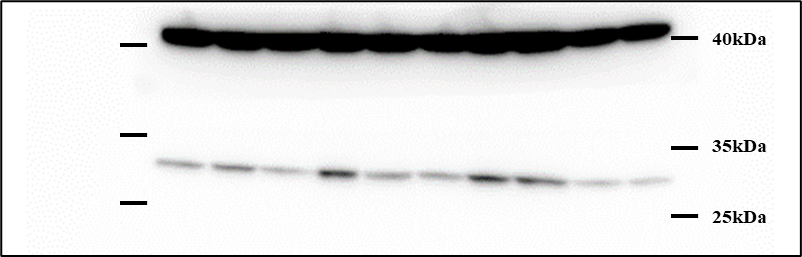


HMOX-1

Used in the manuscript

THGP (+) RBC (-) 1

THGP (+) RBC (+) 1

THGP (+) RBC (+) 2

THGP (-) RBC (-) 2

THGP (-) RBC (+) 1

Another test sample

Another test sample

THGP (-) RBC (+) 2

THGP (+) RBC (-) 2

THGP (-) RBC (-) 1

Membrane photo


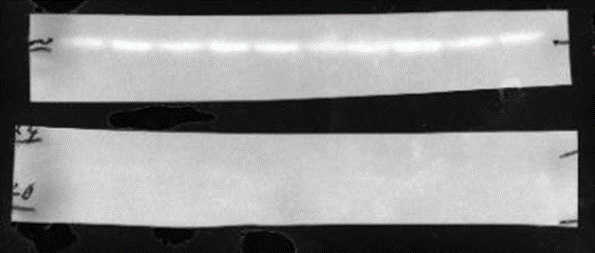


β-ACTIN

HMOX-1

# Table S1 Average values of counted macrophages, phagocytic macrophages and phagocytosed RBCs

The cells of each type were counted according to Materials and Methods subsection 2.3 “Analysis of RBC Phagocytosis by Macrophages”.

|  | **THGP treatment** | **RAW264.7** | **Phagocytic RAW264.7** | **Phagocytosed RBCs** |
| --- | --- | --- | --- | --- |
| **RBC from chow-fed** | **-** | **105.0 ± 4.0** | **50.8 ± 10.2** | **66.2 ± 9.9** |
|  | **+** | **105.5 ± 5.5** | **54.2 ± 5.0** | **77.7 ± 8.1** |
| **RBC from Ge-132-fed** | **-** | **106.6 ± 7.3** | **42.2 ± 10.3** | **52.4 ± 11.5** |
|  | **+** | **104.0 ± 6.8** | **56.3 ± 10.9** | **68.7 ± 14.6** |

※1 The data are presented as the means ± SDs.

# Table S2 Radical-scavenging activity (RSA) of 1 µmol of the pigments derived from heme metabolism, THGP and vitamin C

The RSA was measured according to Materials and Methods subsection 2.9 “Analysis of Radical-Scavenging Activity (RSA) in Feces”.

|  | **(TEs/µmol)** |
| --- | --- |
| **Bilirubin** | **1.90±0.03** |
| **Urobilinogen** | **2.00±0.02** |
| **Stercobilin** | **0.87±0.04** |
| **THGP** | **<0.01** |
| **Vitamin C** | **0.98±0.02** |

※1 The data are presented as the means ± SDs.

※2 The RSA is shown in Trolox equivalents (TEs).
